# Nanozyme-assisted sensitive profiling of exosomal proteins for rapid cancer diagnosis

**DOI:** 10.7150/thno.46568

**Published:** 2020-07-23

**Authors:** Huixia Di, Ze Mi, Yan Sun, Xuehui Liu, Xinzhuo Liu, Ang Li, Ying Jiang, Hongmei Gao, Pengfei Rong, Dingbin Liu

**Affiliations:** 1 Research Center for Analytical Sciences in the College of Chemistry, Tianjin First Central Hospital, State Key Laboratory of Medicinal Chemical Biology, and Tianjin Key Laboratory of Molecular Recognition and Biosensing, Nankai University, Tianjin 300071, China.; 2 Department of Radiology, The Third Xiangya Hospital, Central South University, Changsha, Hunan 410013, China.

**Keywords:** Exosome, nanozyme, protein profiling, cancer diagnosis, nanozyme-assisted immunosorbent assay

## Abstract

The proteins expressed on exosomes have emerged as promising liquid-biopsy biomarkers for cancer diagnosis. However, molecular profiling of exosomal proteins remains technically challenging. Herein, we report a nanozyme-assisted immunosorbent assay (NAISA) that enables sensitive and rapid multiplex profiling of exosomal proteins. This NAISA system is based on the installation of peroxidase-like nanozymes onto the phospholipid membranes of exosomes, thus avoiding the need for post-labelling detection antibodies. The exosomal proteins are determined by a sensitive nanozyme-catalyzed colorimetric assay less than 3 h, without the need for multi-step incubation and washing operations. Using NAISA to profile exosomal proteins from different cell lines and clinical samples, we reveal that tumor-associated exosomal proteins can serve as promising biomarkers for accurate cancer diagnosis in a cooperative detection pattern.

**Methods:** Exosomes were engineered with DSPE-PEG-SH through hydrophobic interaction, and then were assembled with gold nanoparticles (2 nm) to produce Exo@Au nanozyme. The proteins on Exo@Au could be selectively captured by their specific antibodies seeded into a 96-well plate. The immobilized Exo@Au shows peroxidase-like activity to perform colorimetric assays by reaction with 3,3′,5,5′-tetramethylbenzidine (TMB) and H_2_O_2_. The protein levels of exosomes were recorded on a microplate reader.

**Results:** The NAISA platform is capable of profiling multiple exosomal proteins from both cancer cell lines and clinical samples. The expression levels of exosomal proteins, such as CD63, CEA, GPC-3, PD-L1 and HER2, were used to classify different cancer cell lines. Moreover, the protein profiles have been applied to differentiate healthy donors, hepatitis B patients, and hepatic cell carcinoma (HCC) patients with high accuracy.

**Conclusion:** The NAISA nanozyme was allowed to rapidly profile multiple exosomal proteins and could have great promise for early HCC diagnosis and identification of other cancer types.

## Introduction

Exosomes (Exos) are phospholipid membrane-enclosed nanoscale vesicles (30-150 nm in diameter) that are secreted by virtually all types of mammalian cells 1,2. They carry the genetic and protein information from their parent cells to mediate intercellular communication 3,4, and thus actively participate in a variety of pathophysiological processes such as inflammation 5, tissue regeneration 6, and cancer metastasis 7. Recently, Exos have been recognized as promising biomarkers for the liquid biopsy of cancers 8-11. In particular, the levels of specific exosomal proteins correlate well with disease status and can thus be employed as indicators for the early diagnosis of cancer and monitoring of its response to therapy 12,13. Therefore, the capture of exosomal protein information could provide new opportunities for cancer diagnosis.

Rapid analysis of exosomal proteins, however, remains technically challenging due to the small size and chemical complexity of Exos 14,15. Because of their high specificity, immunoassays are commonly employed to analyze exosomal proteins 16,17. Among these, immuno-gold and Western-blot assays represent the gold-standard approaches for identifying specific proteins 13,18, but fail to give quantitative results. Enzyme-linked immunosorbent assay (ELISA) is a classical quantitative method for protein measurement. However, current ELISA methods often require a panel of detection antibodies for the multiplex profiling of exosomal proteins, which can be costly. Moreover, ELISA detection procedures require 5-6 incubation and washing steps, limiting their adoption for rapid analysis 19. Although several advanced assays have recently been utilized for exosomal protein analysis 20-25, these tend to rely on specialized instrumentation and complicated procedures. Thus, the development of a simple but reliable approach for the rapid profiling of multiple exosomal proteins would be highly desirable.

**Figure 1:**
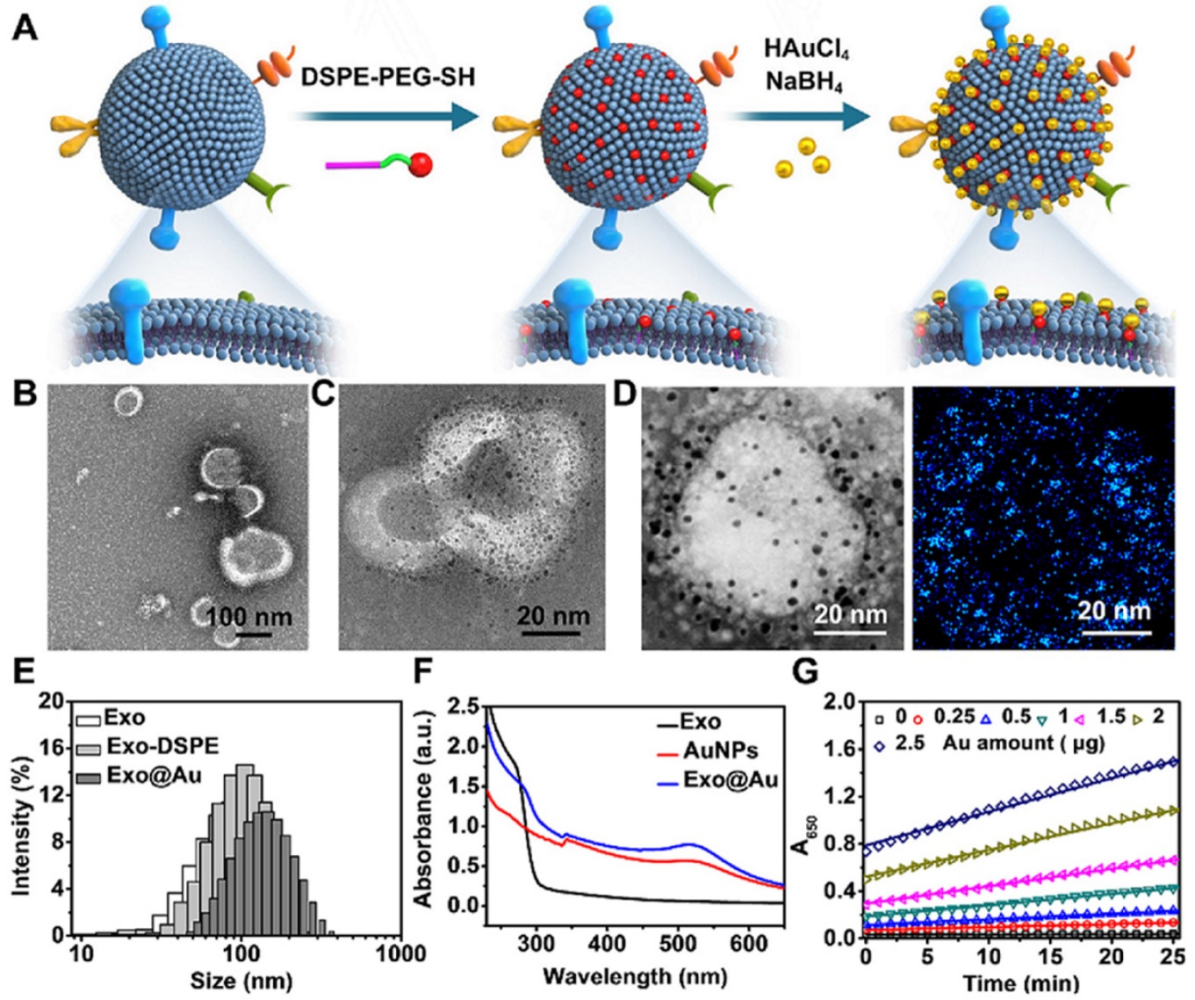
Schematic of the preparation of the gold nanoparticle-decorated exosomes (Exo@Au) and characterization data for the Exo@Au. (**A**) Stepwise preparation of the Exo@Au nanozymes. Representative TEM images of (**B**) the native Exos and (**C**) the Exo@Au nanozymes. (**D**) High-resolution TEM and elemental mapping analysis of Exo@Au. Blue dots represent AuNPs. (**E**) Size distribution of native Exos (Exo, white), DSPE-functionalized Exos (Exo-DSPE, light gray), and Exo@Au (dark gray) as measured by DLS. (**F**) UV-Vis absorption spectra of native Exos (black line), free AuNPs (red line), and Exo@Au (blue line). (**G**) Kinetic measurements of the catalytic activity of Exo@Au with different amounts of Au (0, 0.25, 0.5, 1, 1.5, 2, and 2.5 µg) at 37 °C for 25 min.

Here, a nanozyme-assisted immunosorbent assay (NAISA) that allows sensitive, rapid profiling of multiple exosomal proteins is described. This NAISA system is based on the installation of 2 nm gold nanoparticles (AuNPs) onto the exosomal phospholipid membrane **(Figure 1A)**; these nanoparticles act as peroxidase-like nanozymes with high catalytic efficiency 26-29. The method is performed on a commercially available microplate, whose surfaces have been immobilized with specific capture antibodies for the targeted exosomal protein markers. AuNP-decorated Exos (termed Exo@Au) with the targeted proteins are specifically captured and catalyze a colorimetric reaction; the signal intensity is thus proportional to the level of the targeted exosomal protein. The NAISA technology is superior to conventional immunoassays for exosomal protein analysis because it (i) does not require the use of detection antibodies; (ii) greatly simplifies the detection procedure; and (iii) offers high sensitivity in discriminating different levels of exosomal proteins. The NAISA allows the rapid profiling of multiple exosomal proteins in a variety of cell lines and clinical samples, indicating its great promise for the discovery of cancer biomarkers and early cancer diagnosis.

## Materials and Methods

### Chemicals and instruments

Gold(III) chloride trihydrate (HAuCl_4_•3H_2_O: 99.9%), sodium citrate, hydrogen peroxide solution (30 wt%), and bovine serum albumin (BSA) were purchased from Sigma-Aldrich. Sodium borohydride was obtained from Aladdin (Shanghai, China). Thiol-terminated 1,2-distearoyl-sn-glycero-3-phosphethanolamine-poly (ethylene glycol) [DSPE-PEG-SH] was obtained from Hunan Huateng Pharmaceutical Co., Ltd. (Changsha, China). BCA Protein Quantification Kit was provided by Beyotime Biotechnology (Shanghai, China). Dulbecco's modified eagle medium (DMEM), trypsin and penicillin-streptomycin were all acquired from GIBCO (Grand Island, NY, USA). Anti-CD63 rabbit monoclonal antibody (ab134045), anti-CEA mouse monoclonal antibody (ab4451), anti-GPC-3 rabbit monoclonal antibody (ab66596), anti-PD-L1 mouse monoclonal antibody (ab238697), and anti-HER2 mouse monoclonal antibody (ab16901), Goat anti-Mouse IgG H&L (HRP) (ab205719), and Goat anti-Rabbit IgG H&L (HRP) (ab205718) were purchased from Abcam (Cambridge, MA). Human CD63 ELISA Kit was obtained from Jianglai Biotechnology Co., Ltd. (Shanghai, China). Human PD-L1 ELISA Kit and human HER2 ELISA Kit were purchased from Abcam (Cambridge, MA). Human CEA ELISA Kit and human GPC-3 ELISA Kit were purchased from Abbkine Scientific Co., Ltd. All other reagents used in this text as received did not need any purification.

Cell supernatants, serum samples, and exosome (Exo)-depleted FBS were processed using an ultracentrifuge (L-100 XP, Beckman Coulter). Transmission electron microscopy (TEM) images of AuNPs and Exo@Au were obtained from an electron microscope (Talos F200C). The size distribution and concentration of native Exos were performed by a nanoparticle tracking analyzer (Particle Metrix, Germany). Signal readout of the catalytic reaction was recorded on a microplate reader (Bio-Tek Instrument, Winooski). DLS data and Zeta potential were acquired from a Zetasizer (ZEN1690, Malvern). Au amount was measured by ICP-OES (SpectroBlue). Each protein was identified by an electrophoresis apparatus (JY600C, Zhenzhou, China). The gels of immunoblotting were observed with a gel image system (Tanon-3500). Elemental mapping was performed by a TEM (JEM-2800).

### Synthesis of AuNPs with different sizes

All glass containers and stirring bars were cleared with aqua regia and deionized water before use.

2 nm AuNPs were prepared through the reduction of HAuCl_4_ with NaBH_4_ 30. To 10 mL of aqueous solution was added HAuCl_4_ (a final concentration of 0.25 mM) and trisodium citrate (a final concentration of 0.25 mM) and mixed well. Next, NaBH_4_ solution (0.1 M, 200 µL) was freshly prepared and dropwise added to the solution while stirring for 10 min. The solution became light coral immediately, indicating the successful particle formation. Noteworthy, citrate was used as a capping agent.

8 nm AuNPs were synthesized via Au growth of 2 nm AuNPs 30,31. To a 20 mL bottle, freshly synthesized AuNPs seeds (1 mL) (a final concentration: 0.25 mM) was added and mixed well. Subsequently, the growth solution of HAuCl_4_ (0.25 mM, 9 mL) and cetyltrimethylammoniumbromide (a final concentration of 80 mM) were added to the system. Next, ascorbic acid solution (50 μL, 100 mM) was added while vigorously stirring at room temperature. After stirring for 15 min, the solution turned red and the particles were obtained.

13 nm AuNPs were fabricated through the reduction of HAuCl_4_ with citrates 32. In brief, HAuCl_4_•3H_2_O (39.4 mg) was added into a round-bottom flask containing 100 mL deionized water. The mixture was left to boil with vigorous stirring. Together, trisodium citrate (118 mg, 40 mM) was rapidly added to this system, yielding a wine-red solution of AuNPs. After boiling for 10 min, the heater was removed while stirring was continued for an additional 15 min. The resulting AuNPs were collected after cooling to room temperature.

30 nm AuNPs were synthesized by seed-mediated growth protocol at room temperature 33. Briefly, the 13 nm synthesized AuNPs as seeds were diluted in deionized water (125 mL) with a final concentration of 0.1 nM. Following this step, NH_2_OH (0.2 M, 1 mL) was added aqueous solution rapidly. After stirring for 5 min, HAuCl_4_ (2 mM, 10 mL) was added dropwise for 10 min. The solution turned red gradually with stirring for 30 min and was stabilized with 5% trisodium citrate, indicating the formation of 30 nm AuNPs.

60 nm AuNPs were synthesized based on seed growth method and reduction reaction between HAuCl_4_ with NH_2_OH, which was similar to that of 30 nm AuNPs. Firstly, to 125 mL of as-synthesized AuNP seeds (13 nm, 0.1 nM) was added NH_2_OH (0.2 M, 4 mL) rapidly, followed by stirring for 5 min at room temperature. Next, HAuCl_4_ (2 mM, 40 mL) was added slowly to the mixture with continuous stirring for 30 min. The obtained nanoparticles were stabilized with 5% trisodium citrate, resulting in citrate-capped AuNPs with 60 nm.

### Cell culture

Four cell lines including human liver cell line (LO2), human hepatoma cell line (HepG2), human breast cancer cell line (MCF-7), and human cervical cancer cell line (HeLa) were acquired from the American Type Culture Collection (ATCC). All the cell lines were cultured in DMEM supplemented with 10% FBS and 1% penicillin-streptomycin at 37 °C with 5% CO_2_ until confluence reached 70%. These cells were cultured in fresh DMEM with 10% Exo-depleted FBS for 48 h. The Exos in FBS were previously removed by ultracentrifugation at 100000 g for 16 h. The conditioned cell media were harvested for subsequent Exo isolation.

### Exo enrichment by ultracentrifugation

Cell culture media from the above four cell lines were first centrifuged at 300 g for 10 min, 2000 g for 10 min and 10000 g for 30 min, sequentially removing cells, cellular debris, and microvesicles. Followed by centrifugation, the obtained supernatants were processed through a filter (0.22 µm). Afterward, the resultant solutions were centrifuged at high speed of 100000 g (2×70 min) to result in Exo pellets. All procedures were performed at 4 °C. The Exos were collected and resuspended in sterile PBS (1×) for future use.

Serum samples were provided with the informed consent of volunteers from the Third Xiangya Hospital, Central South University, China. The clinical serum samples obtained from healthy donors (n = 6), hepatitis B patients (n = 12) and hepatocellular carcinoma (HCC) patients (n = 12). All specimens were first processed by low-speed centrifuge as the above steps. Eventually, the resultant samples were diluted with PBS and then subjected to ultracentrifugation at 100000 g (2×70 min) to obtain purified Exos. The enriched Exos were resuspended in sterile PBS (1×) for future use.

### Fabrication of Exo@Au nanozymes

DSPE-PEG-SH was dispersed in anhydrous ethanol as a stock solution at a concentration of 100 µM. To a solution of native Exos (500 µL, 0.2 mg/mL, approximately 4.5×10^9^ particles/mL measured by NTA) was added 2.5 µL of DSPE-PEG-SH (final concentration: 500 nM) under gentle rotation at 4 ^o^C for 20 min. After that, HAuCl_4_ with a final concentration of 1 mM was added to the solution for Au ions recruitment. The resulted mixture was incubated at 37 °C for 40 min. Following this, the residual DSPE lipid and HAuCl_4_ were removed by ultracentrifugation at 100000 g for 30 min, yielding the concentrated Au ion-anchored Exos. The resultant Exos were resuspended in sterile PBS (1×, 200 µL) and then quantified by a BCA kit. Afterward, excess NaBH_4_ (a mild reducing agent, final concentration: 250 µM) was freshly prepared and added to initiate a reduction reaction with gentle stirring. After 5 min, AuNP-decorated Exos (Exo@Au) was fabricated in red color and then stabilized with HS-PEG-OCH_3_ (10 µM, MW: 2000). The Exo@Au nanozymes were measured by UV-Vis spectroscopy.

The average number (*N*) of AuNPs on each Exo was estimated to be around 3500 by the following equation:


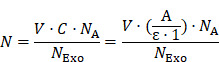


where *V* is the total volume (200 µL) of Exo@Au, *ε* is the molar extinction coefficient of 2 nm AuNPs (4.0×10^6^ L mol^-1^ cm^-1^), and *A* is the absorbance (0.524) of Exo@Au at 510 nm. *C* is the concentration of AuNPs in Exo@Au that is calculated by the Lambert Beers Law. *N_A_* is Avogadro constant (6.02×10²³ mol^-1^), and *N_Exo_* is the number of DSPE-labeled Exos (4.5×10^9^ particles) measured by nanoparticle tracking analysis (NTA).

### Characterization of native Exos and Exo@Au nanozymes

The purified Exos and Exo@Au nanozymes were first verified by TEM imaging. The protocol was performed as follows: 10 µL of Exos (400 μg/mL) or Exo@Au nanozymes (400 μg/mL) was added to carbon-coated copper grids for 3 min, followed by staining with 2% phosphotungstic acid as a contrast agent for 2 min. The residual labelling solution was blotted with a filter paper. After drying, the samples were observed under TEM (Talos F200C) at 100 kV. Together, native Exos were diluted in PBS and then their distribution and concentration were further measured by NTA. Moreover, the Exos could be quantified depending on total proteins through a BCA assay kit as the manufacturer's instructions. Additionally, Exo@Au (400 μg/mL) on copper grids was prepared using the above method and then further confirmed by elemental mapping (TEM, JEM-2800). UV-Vis spectroscopy and ICP-OES assay were allowed to quantify the Au amount of Exo@Au.

### Evaluation of the peroxidase-like activity of AuNPs and Exo@Au nanozymes

To explore the enzymatic property, different sizes of AuNPs (2, 8, 13, 30 and 60 nm) were analysed through a TMB oxidation reaction by H_2_O_2_. The oxidized TMB generated strong signal at a wavelength of 650 nm. The steps were as follows: firstly, TMB (0.4 mg/mL, 50 μL) and H_2_O_2_ (1 M, 50 μL) were spiked to a 96-well plate. Afterward, different sizes of AuNPs were added with the same Au content (2 µg). The total volume of the mixtures was fixed to be 200 µL. Each group was performed three times at 37 °C for 10 min. To improve the detection sensitivity, 2 nm AuNPs-stimulated catalytic reaction was carried out with increasing amounts of H_2_O_2_ (final concentrations ranging from 100 to 500 mM) using this method. Accordingly, the catalytic efficiencies of the as-synthesized Exo@Au nanozymes with different Au amounts were investigated at the optimized concentration of H_2_O_2_ (500 mM). Each group was loaded with the same volume sample of 10 µL, and performed in PBS buffer three times at 37 °C for 10 min. Subsequently, a kinetic study was conducted to measure the catalytic reaction of Exo@Au and AuNPs for 35 min under the same condition. Both groups contained the same Au amount of 2 µg. Taken together, varied concentrations of Exo@Au (corresponding Au amounts: 0.25, 0.5, 1, 1.5, 2, and 2.5 µg) were employed to investigate their kinetics using the above procedures. To further confirm the catalytic stability, Exo@Au (Au amount was 1 µg) was measured at varied time points for 4 days. For all groups, absorbance at 650 nm was collected on a microplate reader.

### Kinetic measurements of Exo@Au

The kinetics of Exo@Au-catalyzed reactions were performed as follows. To a 96-well plate, a series of TMB substrates (0, 0.0104, 0.0208, 0.0416, 0.0832, 0.1248, 0.1664 and 0.2080 mM) and H_2_O_2_ (a final concentration of 250 mM) were added in sodium acetate-citric acid buffer (NaAC-CA, 200 µL, pH = 5.5). For each well, Exo@Au was added with the identical amount of Au (2.5 µg). All groups were incubated at 37 ^o^C. Target signals were recorded on a microplate reader at 650 nm every 30 s. Each case was performed four times. After measurement, the data were analyzed and fitted as a Michaelis-Menten equation to provide Michaelis constant K_m_ and V_max_:


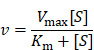


where* v* is reaction velocity, *V_max_* is maximum velocity,* K_m_* is Michaelis constant, [S] is the concentration of the substrate.

### Protein profiling with NAISA

Protein expressions of cell-derived Exos were measured by a nanozyme-assisted immunosorbent assay (NAISA). These proteins involved CD63, CEA, GPC-3, PD-L1, and HER2. To obtain an optimal concentration of Exo@Au for protein detection, Exo@Au from HepG2 cells as a model were sequentially dispersed in sample dilute containing 1% BSA, yielding different concentrations (13.75, 27.5, 55, 110, and 220 μg/mL). Detection procedures were as follows: Exo@Au (100 μL) was added into 96-well plates that had been immobilized with capture antibodies and incubated at 37 °C for 1 h. Following this procedure, the plates were washed four times with washing buffer (1×). Together, for each well, the chromogenic agent of TMB (50 μL from ELISA kit) was added directly, oxidized by H_2_O_2_ (a final concentration of 500 mM) for 15 min, and stopped by 1 M H_2_SO_4_. The wells treated with the same amount of PEG-stabilized AuNPs were set as a blank. A microplate reader was employed to measure the color intensity at 450 nm for each well.

Next, Exo@Au from different cell lines and serum samples (a volume of 600 μL for each sample) were dispersed in sample dilute containing 1% BSA with a final concentration of 220 μg/mL. Exo@Au (100 μL) of each group was added into 96-well ELISA plates and incubated at 37 °C for 1 h. The following procedures were conducted by NAISA as described above.

### Protein profiling with ELISA

Exos from each serum sample was dispersed in sample dilute containing 1% BSA with a final concentration of 220 μg/mL. Each sample (100 μL) was added into 96-well ELISA plates and incubated at 37 °C for 1 h. After incubation, the free samples were removed and the plates were washed four times with washing buffer (1× PBS containing 0.1% BSA). Then, HRP-conjugated secondary antibody (100 μL) was added to ELISA plates and incubated at 37 °C for 1 h. After washing, the plates were treated with TMB (50 μL from ELISA kit) and H_2_O_2_ (a final concentration of 6 mM) for 15 min. The mixtures were finally stopped using 1 M H_2_SO_4_. The wells without the addition of Exos were set as the blank. Signals for each group at 450 nm were recorded on a microplate reader.

### Immuno-gold assay

The proteins (CD63, CEA, GPC-3, PD-L1, and HER2) on Exos were identified by immuno-gold labelling strategy. The model Exos (300 μg/mL) from HepG2 cells were placed onto carbon-coated copper grids for 5 min, followed by immersing into a blocking buffer (1× PBS containing 5% BSA) for 1 h. Afterward, the grids were immediately added into a solution of primary antibody (1:1000) for 2 h at 37 °C. As a control, some grids were not treated with any primary antibody. Next, the immunoblotted samples were washed five times by washing buffer (1×PBS containing 0.1% BSA) and then floated on AuNPs (8 nm)-conjugated secondary antibody solution for 1 h at 37 °C. The grids were rinsed five times and then placed in glutaraldehyde solution (2.5%) for 20 min. After sequentially rinsing with washing buffer and deionized water, the resulting grids were left to dry in the air, stained with a contrast agent of phosphotungstic acid (2%) for 2 min, and observed by a TEM (Talos F200C).

### Western blot analysis

Native Exos and Exo@Au vesicles were lysed by RIPA buffer with an ice bath for 20 min and quantified by a BCA assay. All lysates were separated using sodium dodecyl sulfate-polyacrylamide gel electrophoresis (SDS-PAGE), and then transferred onto nitrocellulose membranes rapidly. The blotting membranes were blocked with 5% non-fat dry milk in TBST buffer (20 mM Tris-HCl, 0.05% Tween-20 and 150 mM NaCl) under 37 °C for 1 h. After that, these membranes were immunoblotted with primary antibodies against the exosomal markers overnight at 4 °C, followed by washing with TBST buffer. A class of protein markers involved CD63, CEA, GPC-3, PD-L1, and HER2. Next, the resulting membranes were incubated with HRP-conjugated secondary antibody for 1 h at 37 °C, and then washed three times with TBST buffer. The western blot images were recorded on a gel image system with the assistance of a chemiluminescence detection kit.

### Dot blotting analysis

To identify the membrane protein expression on Exo surfaces, dot blotting assay was performed to visualize the Exos and Exo@Au *in situ* without protein denaturation. In parallel, the individual PEG-stabilized AuNPs (2 nm) were used as a control. In this test, CD63, CEA, GPC-3, PD-L1, and HER2 were identified by the immunoblotting assay. First, the amounts of native Exos and Exo@Au were quantified and were adjusted to load equal total protein quantities (400 μg/mL). For each dot, 6 μL of the samples was dropped on a nitrocellulose membrane and left to dry in the air. As to both Exo and Exo@Au groups, each group was loaded with the same quantity of total protein (4 μg). For the AuNPs control group, each one was loaded with the same Au contents (2.3 μg) as the Exo@Au group. Thus, the spotted membrane was blocked with 5% non-fat dry milk in TBST buffer (20 mM Tris-HCl, 0.05% Tween-20 and 150 mM NaCl) under 37 °C for 1 h. The primary antibodies, including anti-CD63, anti-CEA, anti-GPC-3, anti-PD-L1, and anti-HER2, were incubated with the samples respectively overnight at 4 °C, followed by incubation with corresponding HRP-conjugated secondary antibodies at 37 °C for 1 h. Eventually, the dots were observed with a chemiluminescence method.

## Results and Discussion

### Evaluation of enzymatic activity of AuNPs

We began our study by synthesizing and optimizing AuNPs with peroxidase-like activity. AuNPs of different sizes (2, 8, 13, 30, and 60 nm) were fabricated, and their physiochemical properties were characterized using transmission electron microscopy (TEM), UV-Vis spectroscopy, and dynamic light scattering (DLS) analysis **(Figure S1)**. Subsequently, peroxidase-like catalytic activity of the AuNPs was analyzed by colorimetry in the oxidation of 3,3′,5,5′-tetramethylbenzidine (TMB) in the presence of H_2_O_2_ 34.The absorbance at 650 nm form oxidized TMB was used as target signal. The results showed that the catalytic performance of the AuNPs was inversely proportional to the particle size **(Figure S2)**, in good agreement with previous reports 35,36. We therefore chose the 2 nm AuNPs as the nanozymes for our NAISA system. We further determined that the colorimetric response was closely related to the concentration of H_2_O_2_ and the nanozyme dose **(Figures S3, S4)**, which is a prerequisite for quantitative detection.

### Physicochemical and catalytic properties of Exo@Au

Next, we attempted to prepare the Exo@Au nanozymes. Model Exos derived from HepG2 (liver cancer cells) were first enriched by ultracentrifugation. The obtained Exos exhibited a typical saucer-shaped morphology **(Figure 1B)**. Nanoparticle tracking analysis (NTA) further revealed that the Exos had a narrow size distribution centered at 106 nm **(Figure S5)**. To immobilize the 2 nm AuNP nanozymes on the surface of the Exos, thiol-terminated 1,2-distearoyl-sn-glycero-3-phosphethanolamine-poly(ethylene glycol) (DSPE-PEG-SH) was inserted into the exosomal membrane via hydrophobic interactions. The DSPE-PEG-SH acted as a handle, as the exposed SH moieties could capture AuNPs tightly via Au-S bonds 37. After incubating the DSPE-PEG-SH-treated Exos with HAuCl_4_ and NaBH_4_, 2 nm AuNPs were formed and uniformly deposited over the vesicle surfaces without altering the Exo morphology **(Figure 1C),** as confirmed by the elemental mapping results **(Figure 1D)**. The average number of AuNPs on each Exo was determined to be approximately 3500 (see details in “Materials and Methods”). In comparison, when native Exos lacking the DSPE-PEG-SH handles were incubated with the same amounts of these Au sources, only a few AuNPs were physically adsorbed onto the exosomal surfaces **(Figure S6)**. The installation of the AuNPs onto the Exos was further verified by their increased hydrodynamic size and Zeta potential, as well as the appearance of a UV-Vis absorption band at ∼510 nm **(Figures 1E,F and S7)**.

The peroxidase-like activity of the Exo@Au nanozymes was evaluated by incubating them in a mixture of TMB (0.1 mg/mL) and H_2_O_2_ (0.5 M) for varying lengths of time. The resulting solutions were initially colorless, but rapidly turned blue, as reflected by the increase of UV-Vis absorbance at 650 nm. As expected, both the absorbance intensity and the reaction rate were positively associated with the amount of Au used to synthesize the 2 nm AuNPs on the Exos **(Figures 1G, S8-12 and Table S1)**. Afterwards, the kinetic study of Exo@Au was performed in **Figure S13**, providing Michaelis constant K_m_ at 0.0387 ± 0.00351 mM and V_max_ at 0.0577 ± 0.00159 mM/min. impressively, the Exo@Au nanozymes maintained their catalytic activity for more than 4 days at ambient temperature** (Figure S14)**, which is vital for the storage, transportation, and practical application of an assay component.

### Profiling of proteins on HepG2 Exos by NAISA

Encouraged by the excellent catalytic performance of the nanozymes 38-40, we then attempted to profile a panel of protein biomarkers on the HepG2-derived Exos using Exo@Au nanozymes based on the NAISA approach. CD63 is a member of the tetraspanin family that is expressed ubiquitously on nearly all cellular Exos 29. Carcinoembryonic antigen (CEA) is commonly used as a clinical biomarker for most types of cancer 41. GPC-3 is a member of the glypican family that is specifically upregulated in hepatocellular carcinoma (HCC) but is not expressed at all in healthy adult livers 42. Human epidermal growth factor receptor 2 (HER2) is typically abundant in breast cancer 22,43. Programmed death-ligand 1 (PD-L1) has recently been recognized as a general cancer biomarker that can be used to predict the response of cancer to immunotherapy 13,24.

**Figure 2:**
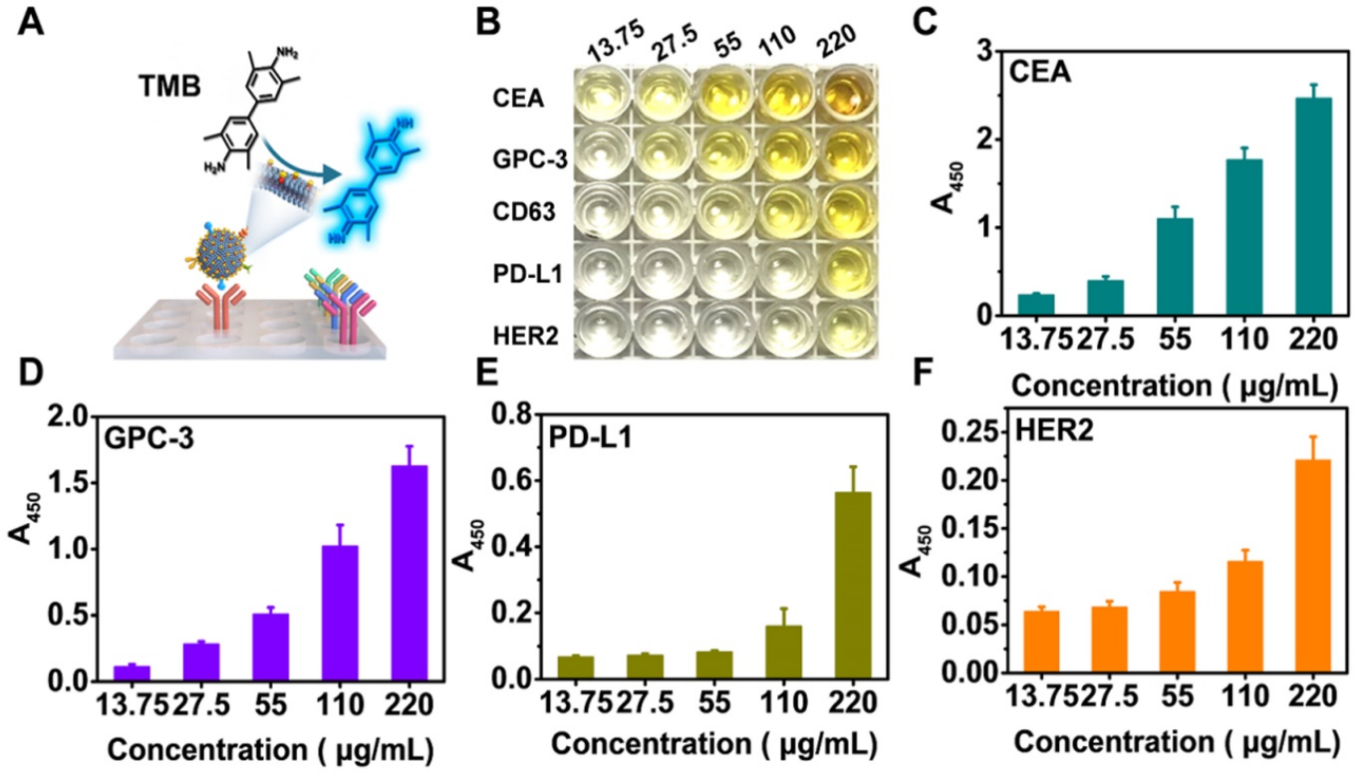
Profiling of a panel of tumor-associated biomarkers on Exos derived from HepG2 cells. (**A**) Schematic illustration of exosomal protein profiling by NAISA. (**B**) Photograph of the colorimetric results for various concentrations of Exo@Au (from 13.75 to 220 µg/mL). Each protein/concentration pair was evaluated in triplicate. Protein levels of (**C**) CEA, (**D**) GPC-3, (**E**) PD-L1 and (**F**) HER2 on HepG2 Exos with different concentrations. Error bars indicated the mean standard deviation of three parallel samples for each case (n = 3).

To perform NAISA, a panel of capture antibodies specifically targeting the above exosomal proteins was immobilized onto the surfaces of microplates (**Figure 2A**). Then, varying doses of Exo@Au (from 13.75 to 220 μg/mL) were incubated in the microplates at 37 °C for 1 h. The Exo@Au nanozymes were PEGylated with SH-PEG-OCH_3_ (MW 2000) to minimize nonspecific adsorption 44. After removing the excess free nanozymes, a mixture of TMB (0.1 mg/mL) and H_2_O_2_ (0.5 M) was added to each well and incubated for 15 min, resulting in blue solutions. When the colorimetric reaction was stopped using 1 M H_2_SO_4_, the solutions turned yellow, and exhibited a target signal at 450 nm. Since CD63 is a general Exo biomarker, we used the level of CD63 as a baseline for comparing the expression of the other four exosomal proteins (**Figure S15**). The results showed that CEA, GPC-3 and PD-L1 were overexpressed in the HepG2 cells, which was reasonable because CEA and PD-L1 are universal cancer biomarker that is up-regulated in most cancer cells, while GPC-3 is a specific HCC biomarker that is highly expressed in HepG2 cells **(Figure 2B-E)**. In contrast, the abundance of HER2 was relatively low in the HepG2 cells, as would be expected based on its irrelevance to HCC (**Figure 2F**). Moreover, expression levels of the four proteins (including CD63, CEA, GPC-3 and PD-L1) for diverse samples were fitted as concentration-response curves, resulting in a good linear pattern (**Figure S16**). Overall, the NAISA system can differentiate the levels of the different exosomal proteins and does not require post-labelling detection antibodies, which not only reduces its cost, but also greatly simplifies the detection procedure.

### Identification of exosomal proteins by immunoblotting assays

**Figure 3:**
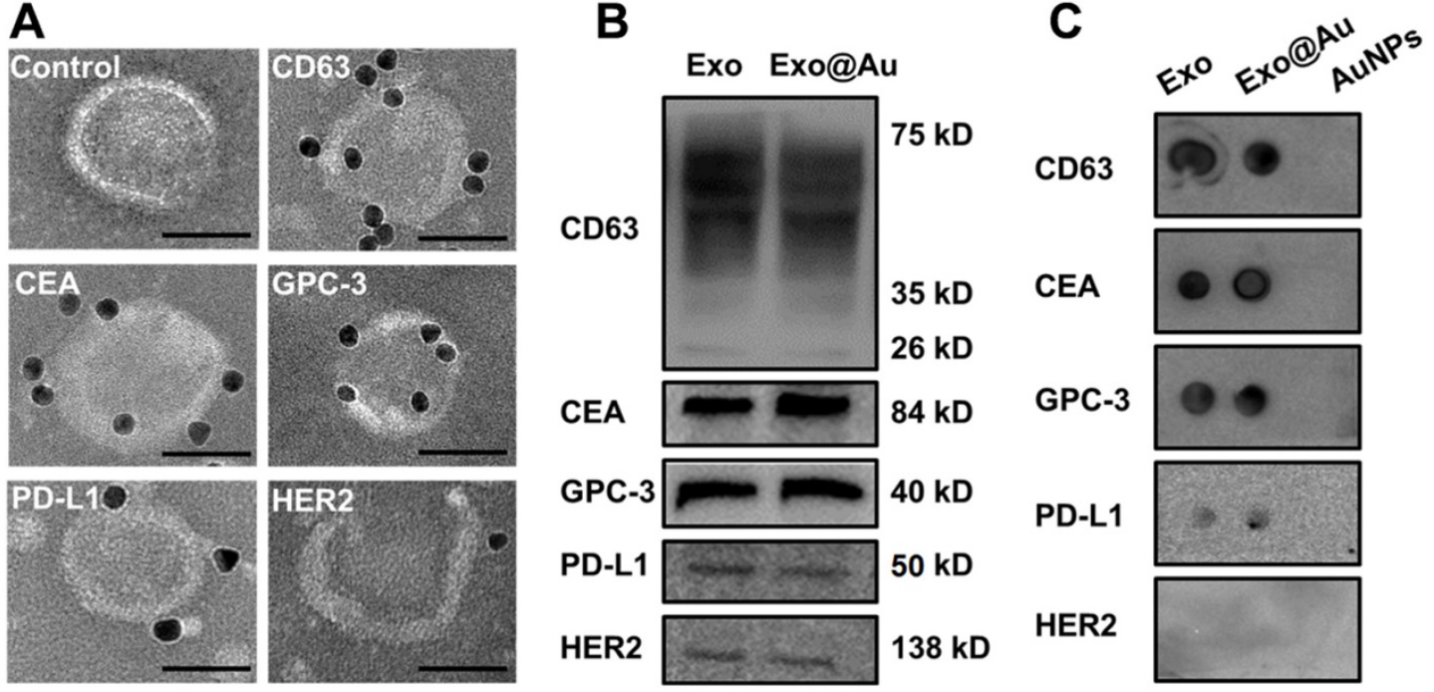
Identification of the five proteins on HepG2 Exos by immunoblotting assays. (**A**) Representative TEM images of HepG2 Exos labeled with different immuno-gold antibodies: Anti-CD63, anti-CEA, anti-GPC-3, anti-PD-L1, and anti-HER2. A mixture of Exos and pure AuNPs was used as a control. The labeled AuNPs had a mean size of 8 nm. Scale bar: 50 nm. (**B**) Determination of the five exosomal biomarkers by Western-blot analysis. Each group was loaded with the same quantity of total denatured proteins (15 µg). (**C**) Dot blotting analysis of the tested biomarkers on native Exos and Exo@Au nanozymes without protein denaturation. PEG-stabilized AuNPs (2 nm) were used as a control.

To validate the NAISA results, immuno-gold assays were performed to determine whether these protein biomarkers were situated on the HepG2 exosomal membranes. After individually incubating antibody-labeled AuNPs (8 nm) with the HepG2 Exos, multiple immuno-AuNPs against CD63, CEA, and GPC-3 were observed on the membranes of HepG2 Exos, while much smaller numbers of immuno-AuNPs against PD-L1 and HER2 were found **(Figure 3A)**. As a control, the Exos were incubated with the naked AuNPs alone; no NPs were found on the exosomal membranes. The average numbers of immuno-AuNPs against CD63, CEA, GPC-3, PD-L1, and HER2 on each Exo (n = 10) were estimated to be 8.5, 7.7, 7.0, 4.2, and 1.7 **(Figure S17)**. In parallel, a Western blot was conducted to confirm the abundance of these biomarkers in the Exo population. Intense bands for CD63, CEA, and GPC-3 and weak bands for PD-L1 and HER2 were clearly observed for both HepG2 Exos and Exo@Au nanozyme lysates** (Figure 3B)**.

We wondered whether the installed AuNPs would hamper interactions between the exosomal proteins and their specific antibodies. Dot blotting assays were carried out on the Exo@Au nanozymes *in situ*, and the results were compared with those obtained using native HepG2 Exos. PEG-functionalized AuNPs (2 nm) were blotted as a control. The native Exos and Exo@Au nanozymes showed similar results to the Western-blot analysis **(Figure 3C)**. The control group did not generate any immunoblot dots. These results implied that the installation of the nanozymes had a negligible influence on the recognition of the exosomal proteins by their capture antibodies.

### Profiling of exosomal proteins from multiple cell lines

**Figure 4:**
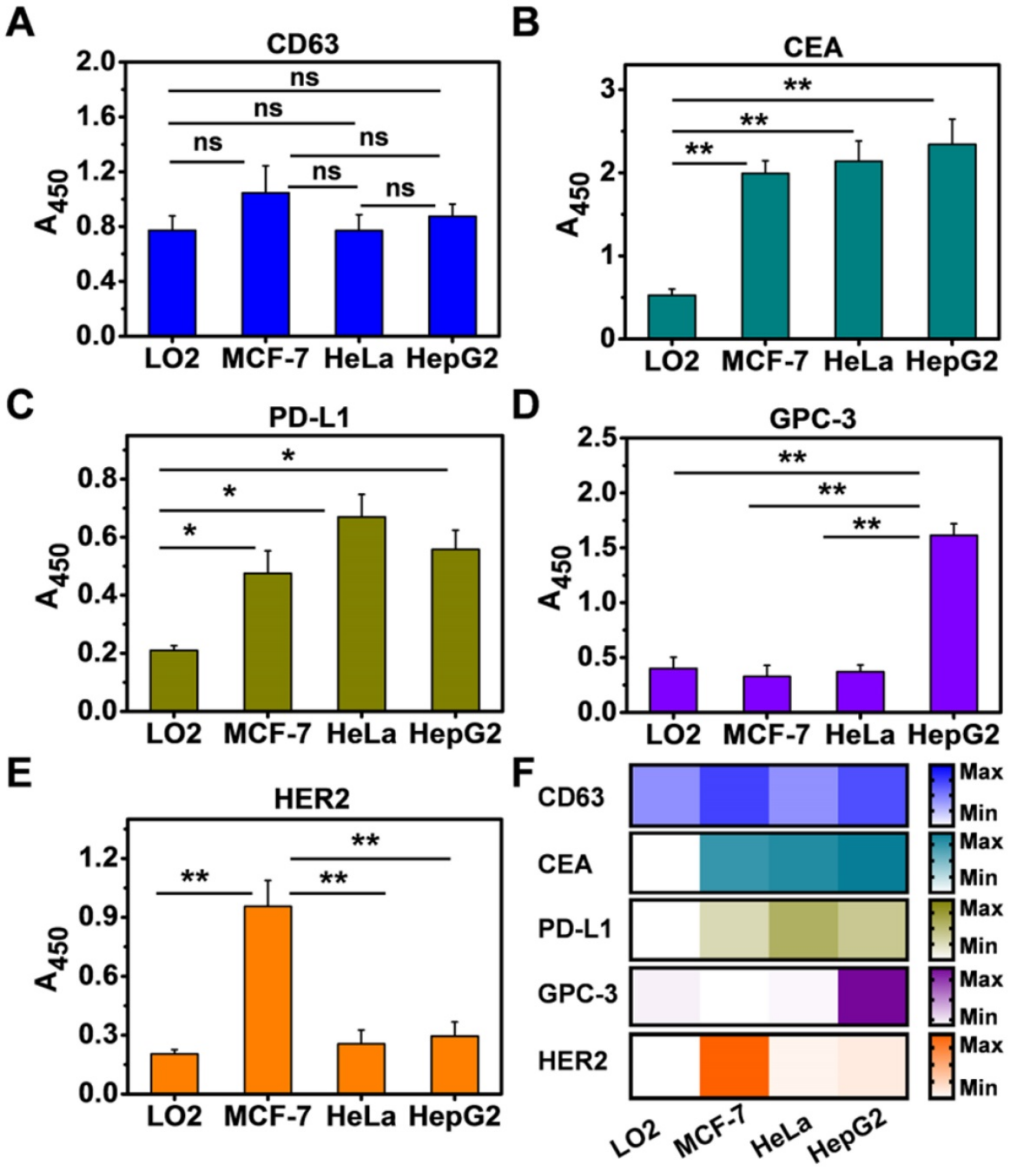
Simultaneous profiling of five exosomal proteins from four cell types, including LO2, HepG2, MCF-7, and HeLa. The expression levels of (**A**) CD63, (**B**) CEA, (**C**) PD-L1, (**D**) GPC-3, and (**E**) HER2 were profiled using the NAISA platform. Each test was performed in triplicate using three parallel samples. The Exo concentration was fixed at 220 µg/mL. (**F**) Expression levels of the five biomarkers on the four types of cellular Exos summarized as a heat map. Error bars indicated the mean standard deviation of three parallel samples for each case (n = 3). *P < 0.05, **P < 0.01, ns = no significant difference.

Having demonstrated that NAISA can serve as a simple, sensitive, and specific platform for analyzing multiple exosomal proteins, we sought to extend our strategy to the molecular screening of exosomal proteins from other cell types, namely, LO2 (normal human hepatic cells), MCF-7 (human breast cancer cells), and HeLa (human cervical cancer cells). For this test, Exos were first enriched from culture media containing the respective cell types, verified by TEM **(Figure S18)**, and decorated with 2 nm AuNPs. The five biomarkers were simultaneously measured using NAISA; the concentration of each kind of Exo@Au nanozymes was fixed at 220 μg/mL. As expected, CD63 was present on all four Exo types **(Figure 4A)**; CEA and PD-L1 were positively expressed on the cancer cell Exos but were present in significantly lower levels in the noncancerous LO2 Exos **(Figure 4B,C)**. GPC-3 and HER2 were specifically up-regulated on the HepG2 and MCF-7 Exos, respectively **(Figure 4D,E)**. The results indicate that exosomal GPC-3 and HER2 could independently serve as specific indicators for liver and breast cancer diagnosis **(Figure 4F)**. These results suggest that NAISA could provide a new platform for the rapid profiling of exosomal proteins secreted from a variety of cell lines, and thus shows great promise for the discovery of new cancer biomarkers.

### Profiling of exosomal proteins from clinical samples

Based on the results of the above investigations, we trust that NAISA could be used in clinical diagnosis. HCC is one of the most common malignant tumors, with more than 782,000 new cases and 746,000 deaths annually 45. Most HCC cases occur in patients infected with hepatitis B, especially in developing countries 46. The ability to discriminate HCC from hepatitis B infection at its earliest stage would be highly beneficial to HCC management. In recent years, exosomal proteins have emerged as effective biomarkers and could provide new opportunities for cancer diagnosis 8,9,20. Therefore, we hypothesized that the NAISA system could exploit exosomal protein profiling to identify HCC owing to the high abundance of Exos circulating in the blood.

**Figure 5:**
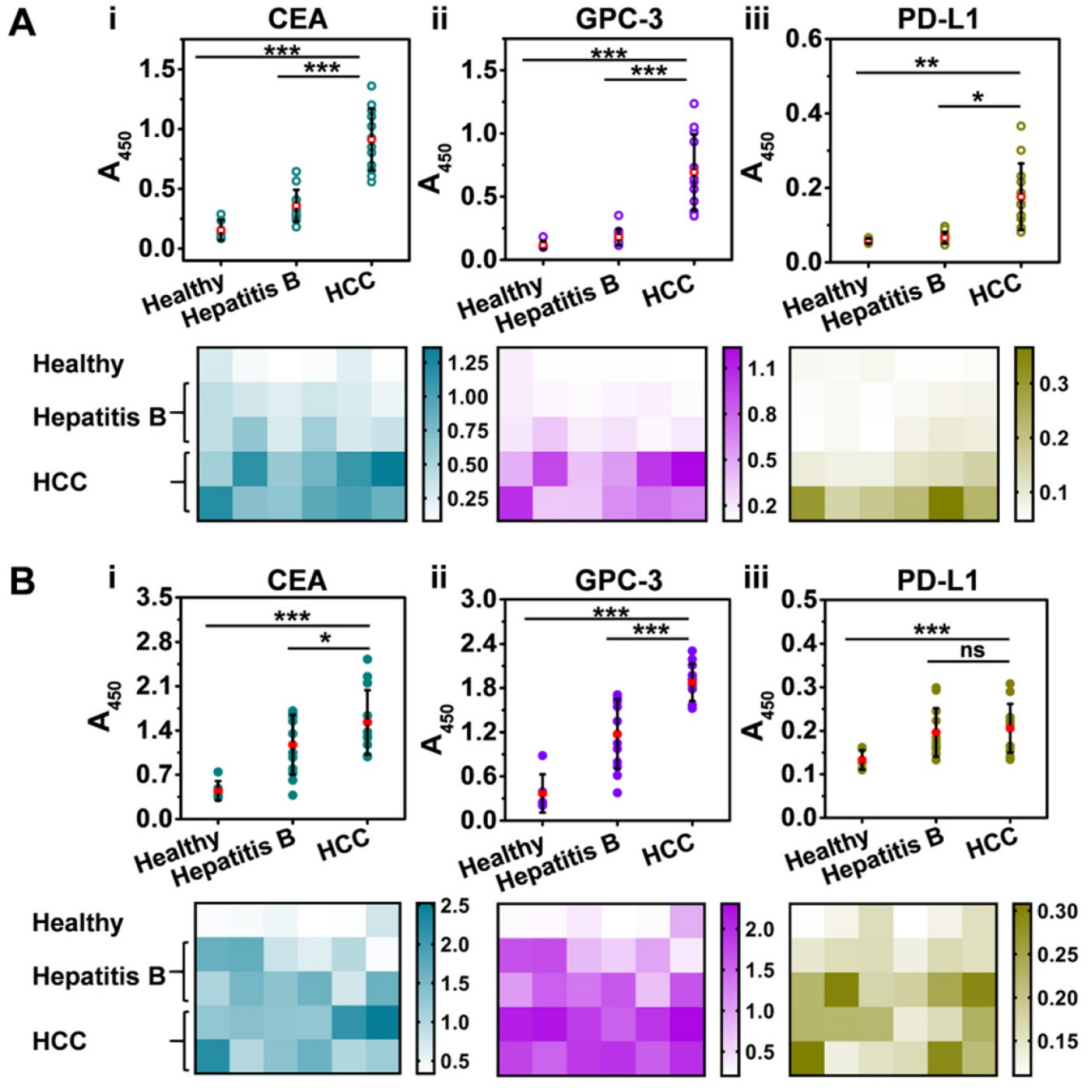
Profiling of tumor-associated exosomal proteins in clinical serum samples collected from healthy donors (n = 6), patients with hepatitis B (n = 12), and patients with HCC (n = 12). Discrimination of healthy, hepatitis B, and HCC patients by (i) CEA, (ii) GPC-3 and (iii) PD-L1 profiling using (**A**) NAISA and (**B**) ELISA. Heat maps of the protein profiles for each sample obtained using (A) NAISA (B) and ELISA. *P < 0.05, **P < 0.01, ***P < 0.001, ns = no significant difference.

We first isolated Exos from clinical serum samples that were collected from healthy donors (n = 6), patients with hepatitis B (n = 12), and patients with HCC (n = 12) with the informed consent of the volunteer donors **(Tables S2,S3)**. Subsequently, the Exos were decorated with 2 nm AuNPs to yield Exo@Au nanozymes. NAISA was conducted to determine the expression levels of the five proteins on the Exos. As indicated in **Figure 5A**, for the tumor-associated protein groups (CEA, GPC-3, and PD-L1), the average signal intensity gradually increased in the order: healthy < hepatitis B < HCC, demonstrating the initiation and progression of HCC. Protein levels for each sample were also summarized as heat maps. Impressively, both exosomal CEA and GPC-3 could effectively differentiate HCC samples from other samples (**Figure 5A**, i and ii, P < 0.001; two-tailed t-test). The results were confirmed using ELISA **(Figure 5B)**, indicating the great potential of these exosomal proteins as an effective biomarker for the early diagnosis of HCC. Moreover, the NAISA platform enabled unambiguous discrimination between donors with hepatitis B and those with HCC, which was in full agreement with the clinical results. In comparison, conventional ELISA was unable to distinctly differentiate the two groups, especially for CEA (**Figure 5B**, i, P < 0.05) and PD-L1 detection (**Figure 5B**, iii, no significance). Accordingly, CD63 (a positive control) and HER2 (a negative control) profiling were conducted and summarized in **Figure S19**. Notably, principal component analysis (PCA) of the NAISA data allowed clear discrimination of HCC samples from the hepatitis B and healthy ones using the protein (CEA, GPC-3 and PD-L1) signatures (**Figure S20A**). However, the ELISA result was inferior to that obtained using NAISA (**Figure S20B**). These encouraging demonstrated that, the NAISA system provided high discrimination capability for cancer diagnosis. Using the NAISA technique, cooperative detection of diverse disease-associated exosomal proteins is expected to become a powerful tool for the early diagnosis and monitoring. Additionally, to compare the performance for protein profiling, the characteristics of the existing methodologies were summarized in **Tables S4.**

## Conclusion

In summary, we have developed a simple yet sensitive NAISA platform that can rapidly profile multiple exosomal proteins. Unlike conventional ELISA, which relies on post-labelling detection antibodies, the NAISA method does not require detection antibodies. Instead, the labels (2 nm AuNP nanozymes) are installed directly on the exosomal phospholipid membrane, thus greatly reducing the cost of exosomal protein profiling and simplifying operation procedures. This NAISA platform enabled the profiling of exosomal protein patterns from different cell lines. Future work will focus on analyzing clinical samples to verify the reliability of exosomal proteins in the early diagnosis, molecular staging, and therapeutic monitoring of cancer, as well as extending the NAISA method to the detection of other exosomal proteins relevant to a wide variety of diseases.

## Supplementary Material

Supplementary figures and tables.
