# Supplementary material for: Nanozyme-assisted sensitive profiling of exosomal proteins for rapid cancer diagnosis
Source: Theranostics. 2020 Jul 23;10(20):9303–14. doi: 10.7150/thno.46568 (PMC7415820; doi:10.7150/thno.46568)
Supplement: Supplementary file 1 — Supplementary figures and tables. [file thnov10p9303s1.pdf]

Supplementary Information

Nanozyme-assisted sensitive profiling of exosomal proteins for rapid cancer diagnosis

Huixia Di[1], Ze Mi[2], Yan Sun[1], Xuehui Liu[1], Xinzhuo Liu[1], Ang Li[1], Ying Jiang[1], Hongmei Gao[1]*, Pengfei Rong[2]*, Dingbin Liu[1]*

1. Research Center for Analytical Sciences in the College of Chemistry, Tianjin First Central Hospital, State Key Laboratory of Medicinal Chemical Biology, and Tianjin Key Laboratory of Molecular Recognition and Biosensing, Nankai University, Tianjin 300071, China
2. Department of Radiology, The Third Xiangya Hospital, Central South University, Changsha, Hunan 410013, China

*E-mail: ghm182@163.com; rongpengfei66@163.com; liudb@nankai.edu.cn

Supplementary Figures and Tables

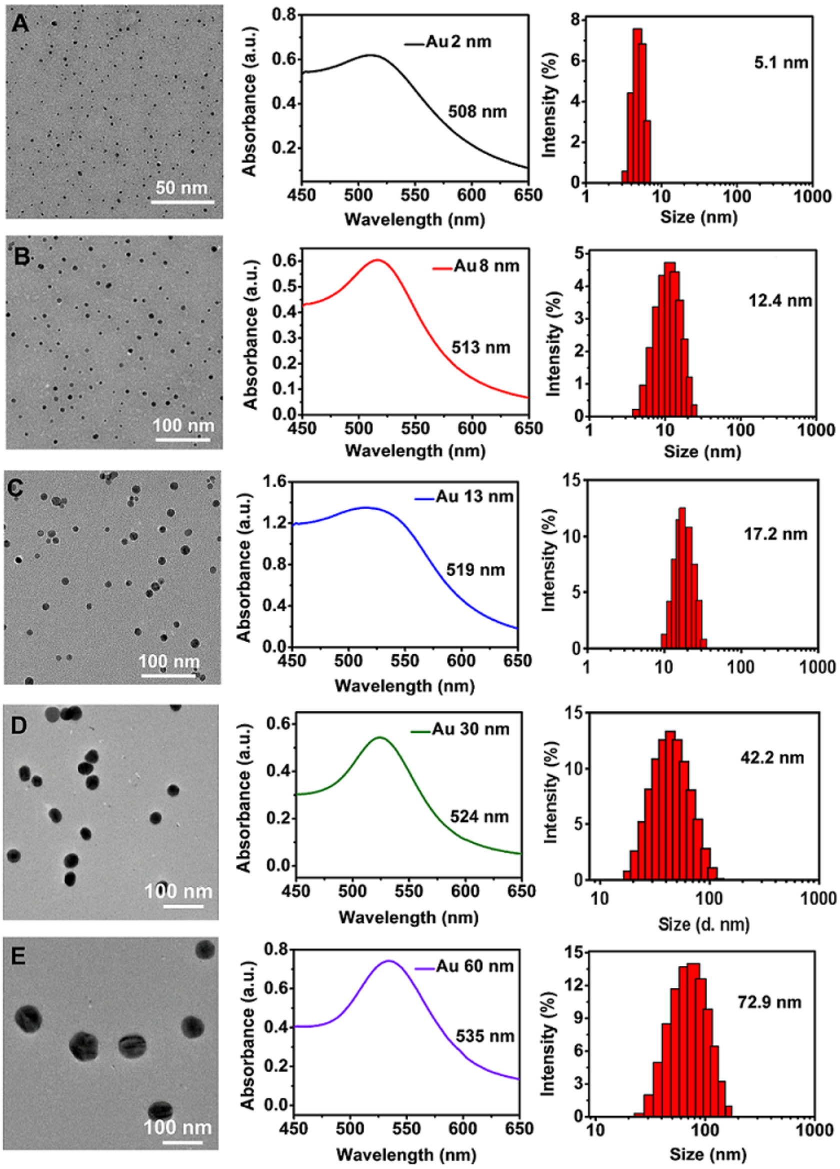

Figure S1. Different sizes of AuNPs that were characterized by TEM (the first column), UV-Vis spectroscopy (the second column), and DLS analysis (the third column). (A) 2 nm; (B) 8 nm; (C) 13 nm; (D) 30 nm; and (E) 60 nm.

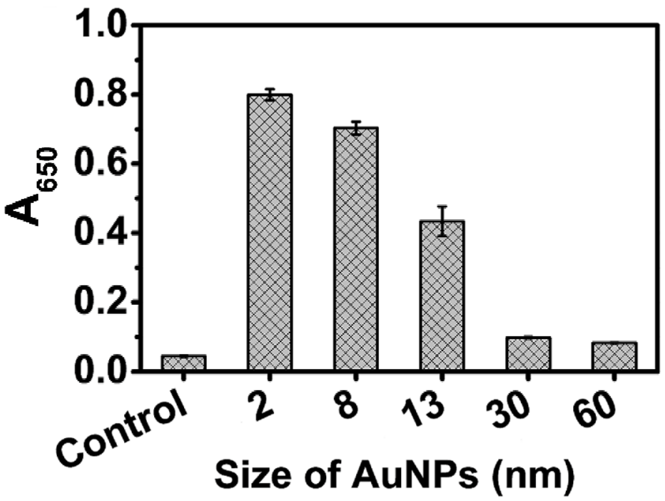

Figure S2. Absorbance at 650 nm (A_{650}) of different sized AuNPs (ranging from 2 nm to 60 nm) in the presence of TMB (0.1 mg/mL) and H_2O_2 (0.25 M). The Au amount for each size of AuNPs was identified to be 2 μg. Error bars represent three parallel samples.

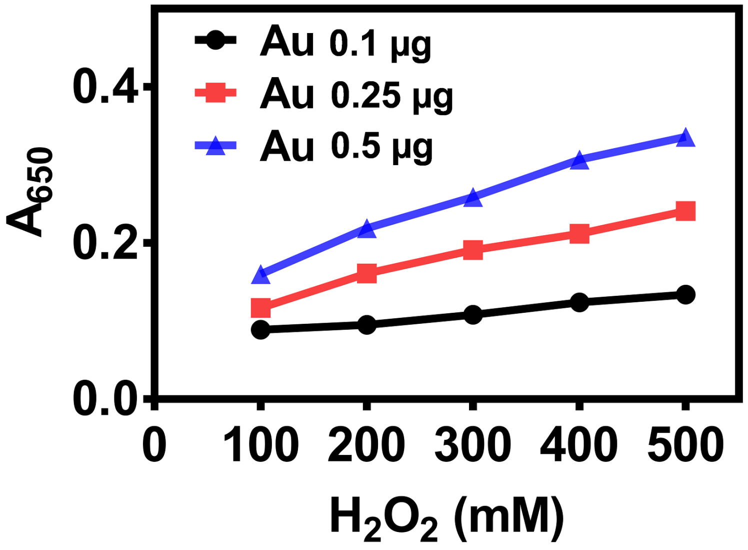

Figure S3. Absorbance at 650 nm (A_{650}) of the catalytic reaction stimulated by AuNPs (2 nm) under varied Au amounts (0.1, 0.25, and 0.5 μg) in the presence of different concentrations of H_2O_2 (from 100 to 500 mM) and TMB (0.1 mg/mL). The mixtures were incubated for 10 min at 37 ^{o}C before collecting the absorbance under a microplate reader.

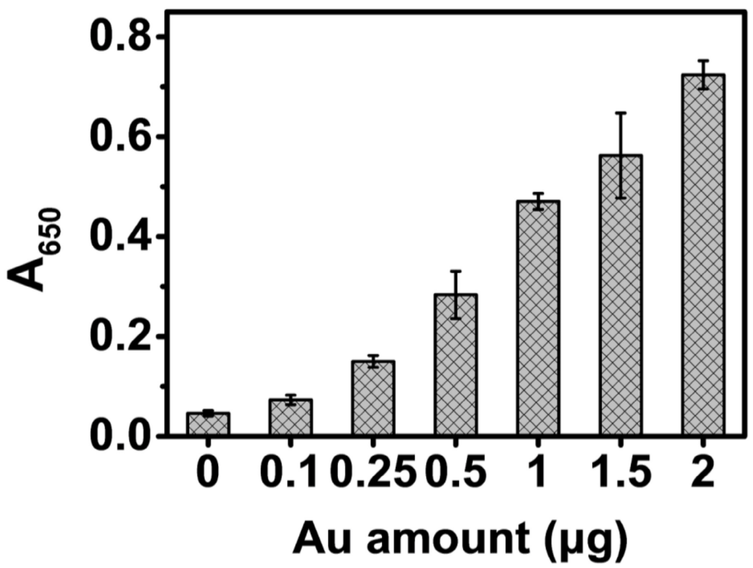

Figure S4. Absorbance at 650 nm (A_{650}) of the catalytic reaction stimulated by AuNPs (2 nm) under varied Au amounts (ranging from 0.1 to 2 μg) in the presence of H_2O_2 (0.5 M) and TMB (0.1 mg/mL). The mixtures were incubated for 10 min at 37 ^oC before collecting the absorbance under a microplate reader. Error bars represent three parallel samples.

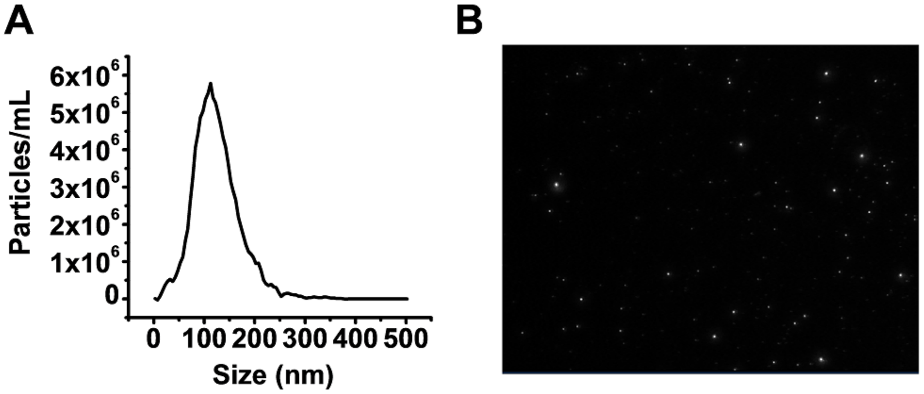

Figure S5. (A) Size distribution and concentration of native Exos were measured by nanoparticle tracking analysis (NTA). (B) A representative photograph of Exos observed using NTA.

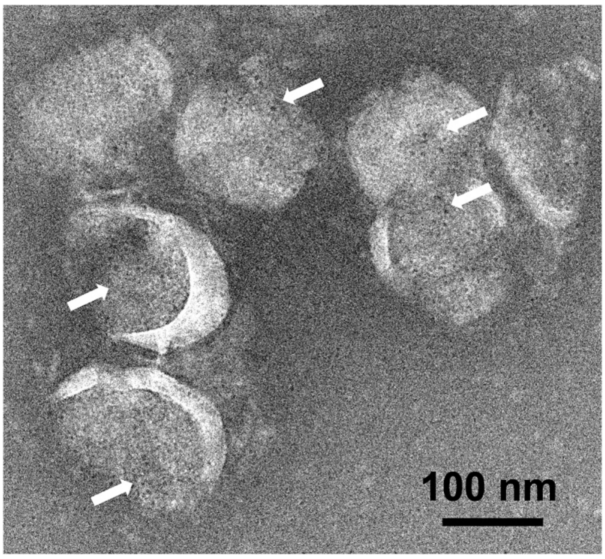

Figure S6. A TEM image of Exo@Au synthesized without DSPE-PEG-SH engineering.

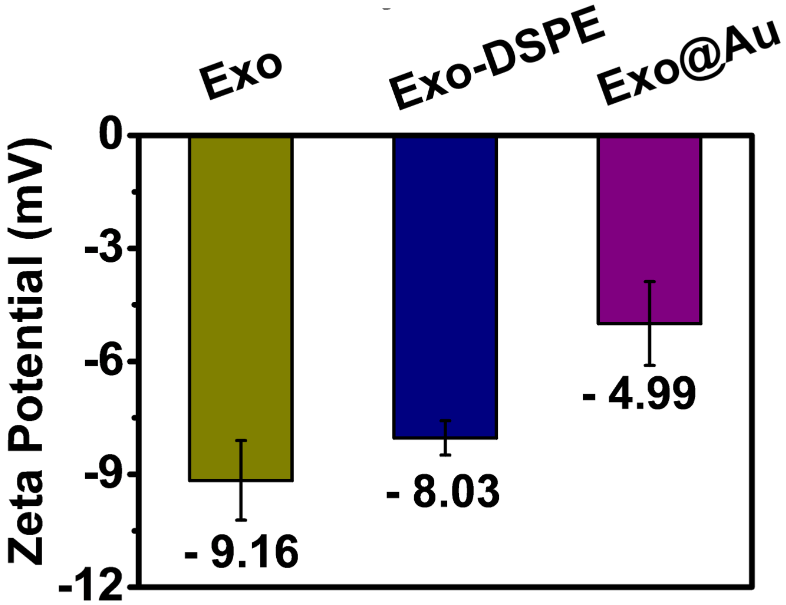

Figure S7. Zeta potential analysis of these vesicles, including native Exos, Exo-DSPE, and Exo@Au. Each group was performed with three parallel samples.

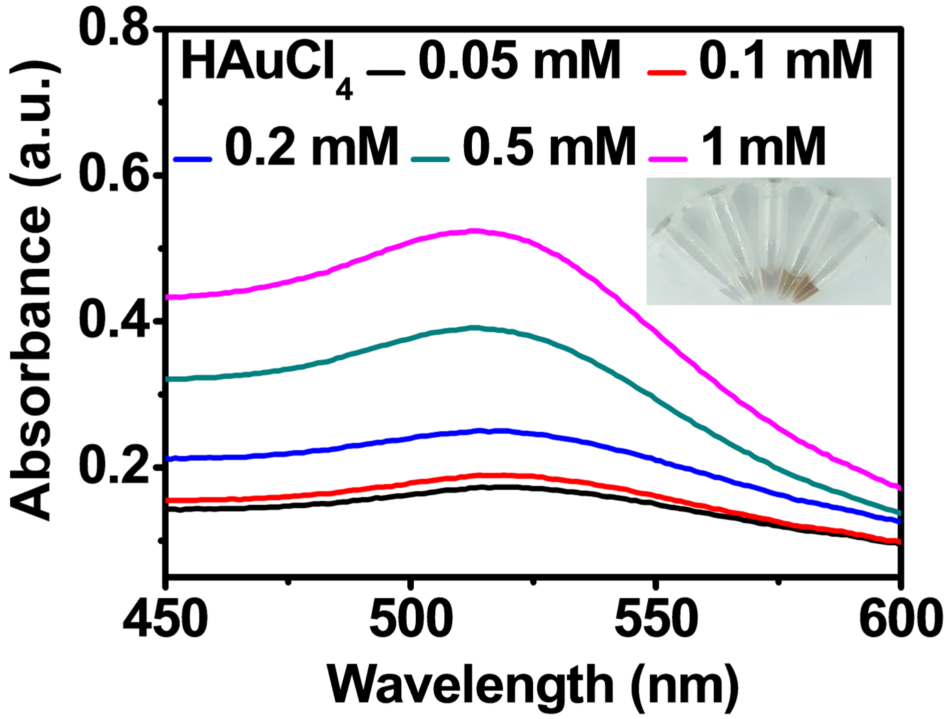

Figure S8. UV-Vis absorption spectra of Exo@Au produced from varied concentrations of $HAuCl_4$ (0.05, 0.1, 0.2, 0.5, and 1 mM) and $NaBH_4$ (250 µM). Inset: corresponding colored picture of the Exo@Au. From left to right: 0.05, 0.1, 0.2, 0.5, and 1 mM.

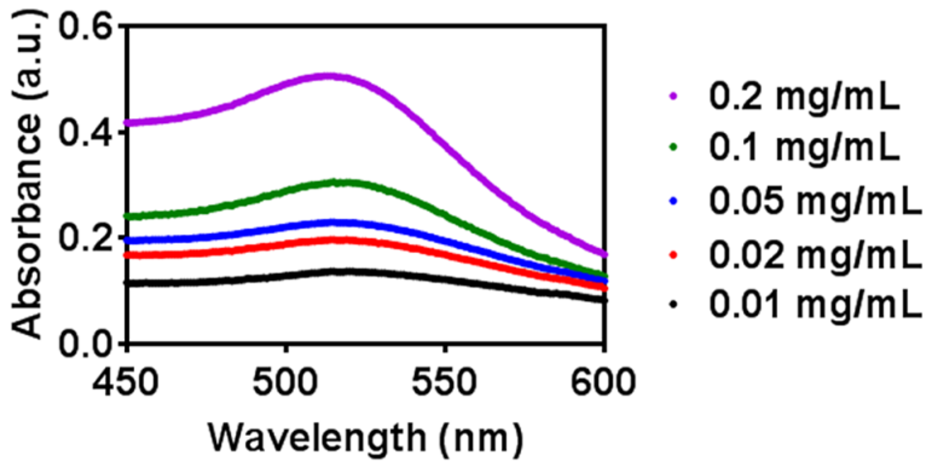

Figure S9. UV-Vis absorption spectra of Exo@Au produced from varied concentrations of Exos (0.01, 0.02, 0.05, 0.1, and 0.2 mg/mL), $HAuCl_4$ (1 mM) and $NaBH_4$ (250 µM).

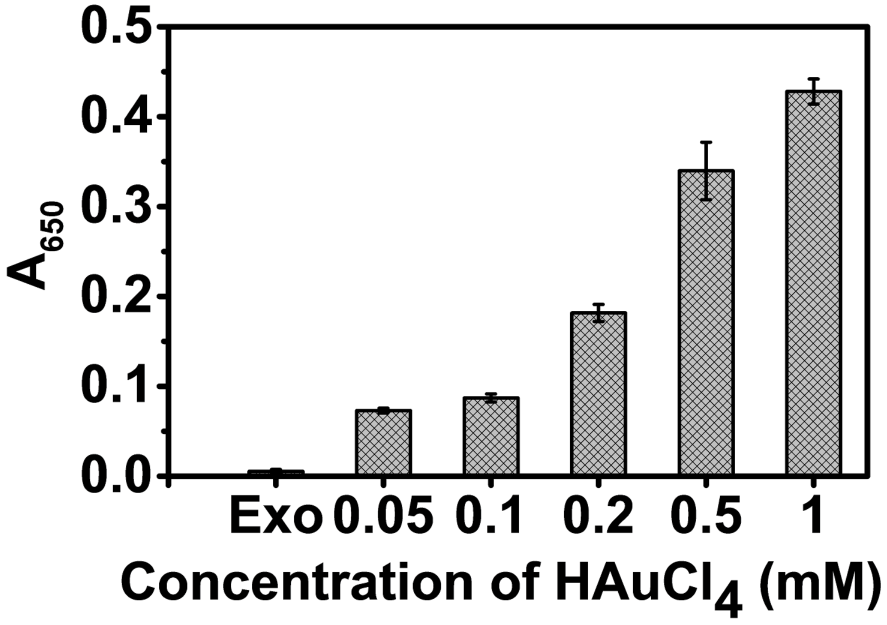

Figure S10. Absorbance at 650 nm (A_{650}) of the resultant Exo@Au nanozyme (prepared with different $HAuCl_4$ concentrations: 0.05, 0.1, 0.2, 0.5, and 1 mM) in the presence of TMB (0.1 mg/mL) and H_2O_2 (0.5 M). Each group was performed with three parallel samples.

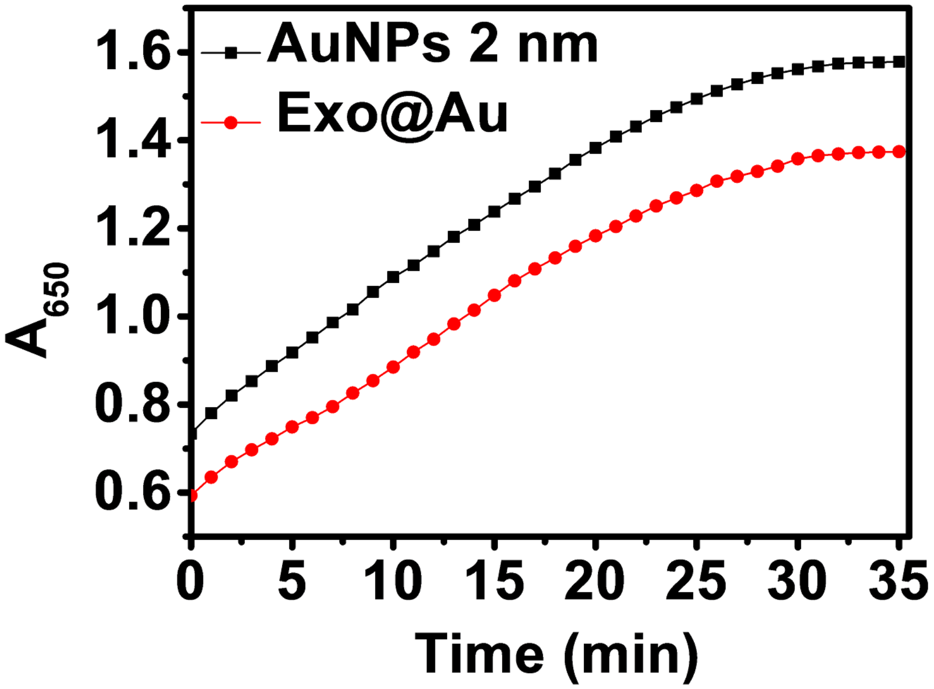

Figure S11. A kinetic study to measure the catalytic property of Exo@Au and AuNPs at 37 °C for 35 min. The both groups were added with the same Au amount (2 μg).

Table S1. Linear curves were fitted from a kinetic study of Exo@Au with varied concentrations.

Exo@Au (Au content: μg)	Linear fitting equation	R^2
0.25	Y = 0.00253 X + 0.0706	0.996
0.5	Y = 0.00478 X + 0.113	0.999
1	Y = 0.00958 X + 0.185	0.997
1.5	Y = 0.0147 X + 0.294	0.997
2	Y = 0.0225 X + 0.522	0.992
2.5	Y = 0.0284 X + 0.786	0.988

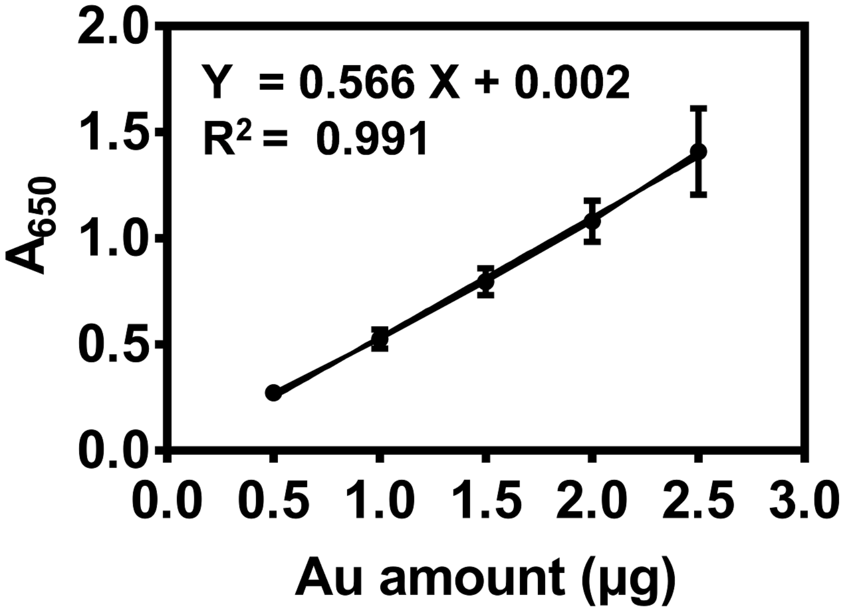

Figure S12. The absorbance at 650 nm of Exo@Au-catalytic products collected at 25 min, resulting in a linear pattern ranging from 0.5 to 2.5 μg. Each group was performed with three parallel samples.

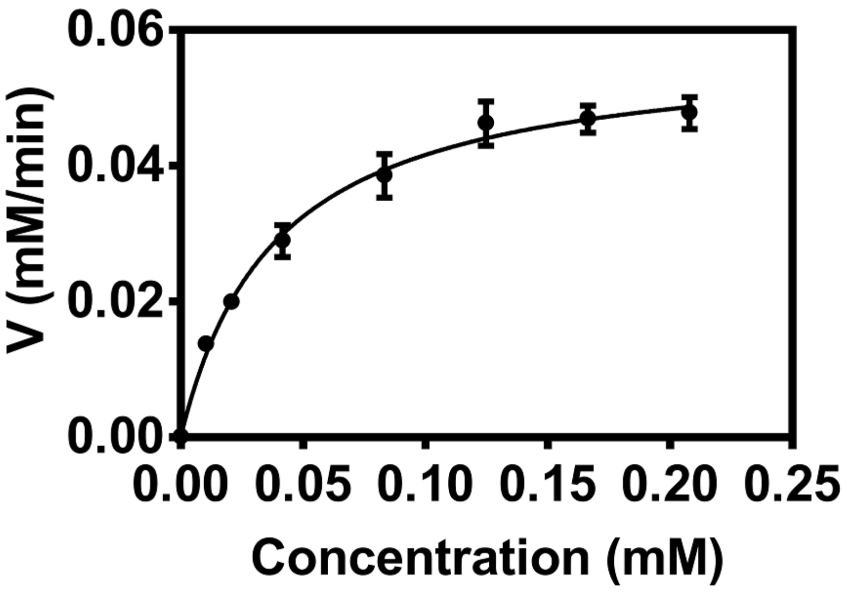

Figure S13. A kinetic study of the catalytic reaction of Exo@Au (Au: 2.5 μg) with different concentrations of TMB (0, 0.0104, 0.0208, 0.0416, 0.0832, 0.1248, 0.1664, 0.2080 mM) in NaAc-CA buffer (pH = 5.5) at 37 °C. The curves were fitted with Michaelis-Menten equation. Error bars indicated the mean standard deviation of four parallel samples for each case (n = 4).

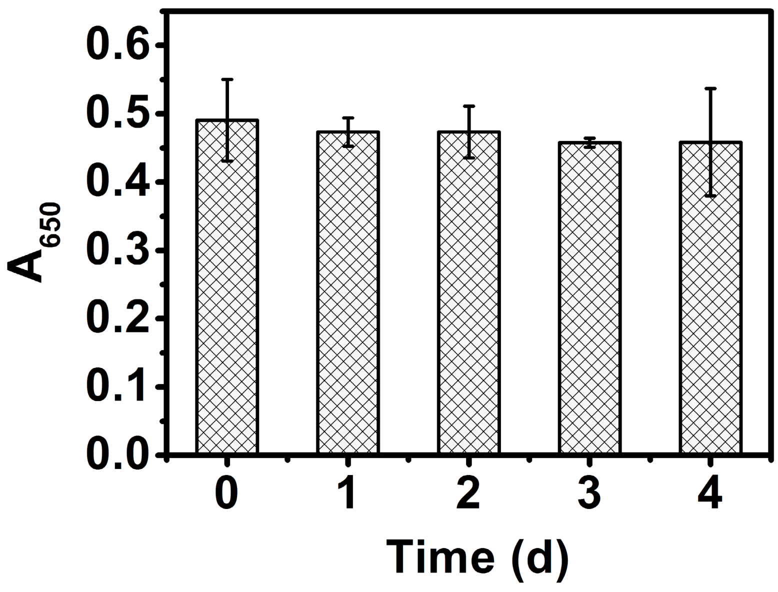

Figure S14. The catalytic performance of Exo@Au (Au content: 1 μg) was measured at varied time points for 4 days. Each group was performed with three parallel samples.

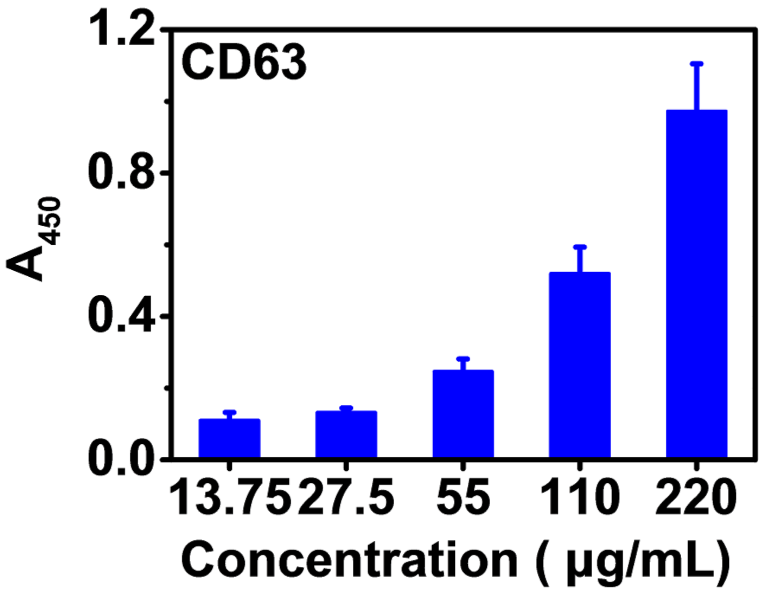

Figure S15. Protein levels of CD63 on HepG2 Exos with different concentrations by NAISA. Error bars indicated the mean standard deviation of three parallel samples for each case (n = 3).

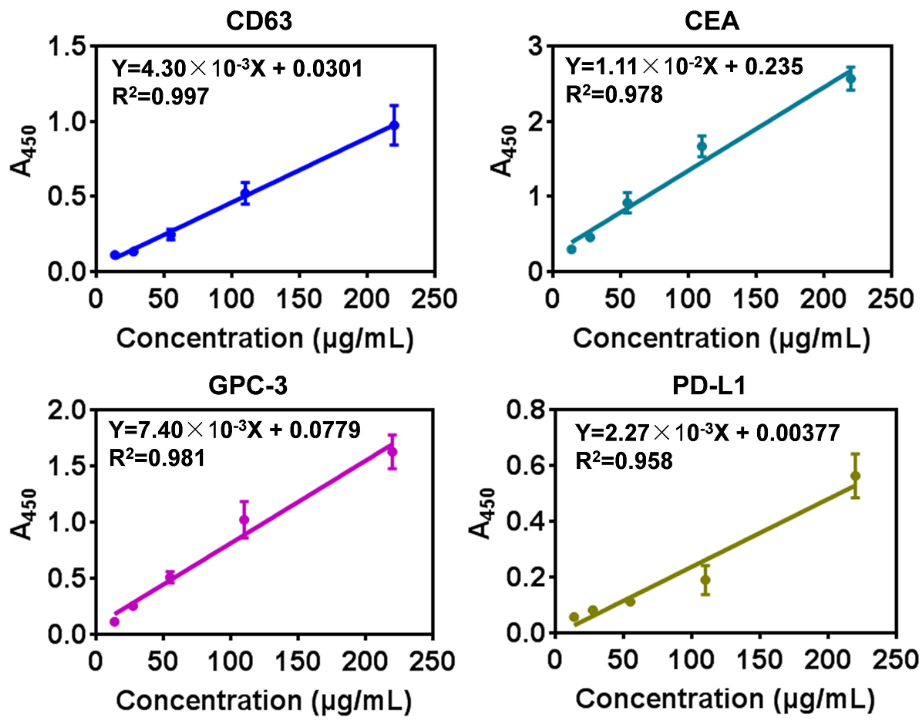

Figure S16. Protein levels of (A) CD63, (B) CEA, (C) GPC-3, and (D) PD-L1 on HepG2 Exos were fitted as concentration-response curves, resulting in a good linear pattern. Error bars indicated the mean standard deviation of three parallel samples for each case (n = 3).

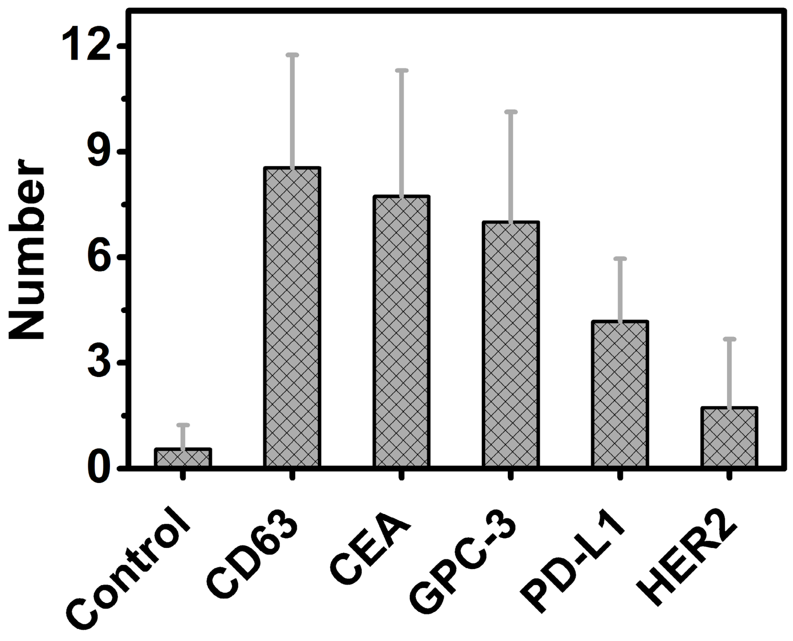

Figure S17. Statistical analysis of AuNP numbers on each Exo for specific protein markers in the immuno-gold assays. A mixture of Exos and pure AuNPs served as a control. Error bars represent the mean standard deviation of multiple Exos (n = 10) for each case.

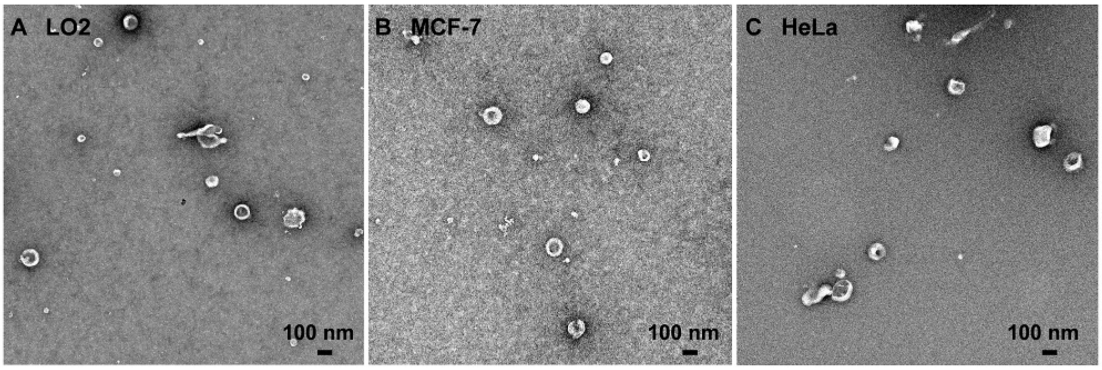

Figure S18. Representative TEM micrographs of (A) LO2 Exos, (B) MCF-7 Exos, and (C) HeLa Exos.

Table S2. Basic information of the healthy donors and hepatitis B patients.

Healthy Characteristic	Gender	Age	Hepatitis B Characteristic	Gender	Age
Donor 1	Male	31	Patient 1	Male	47
Donor 2	Female	51	Patient 2	Female	33
Donor 3	Male	29	Patient 3	Male	50
Donor 4	Male	27	Patient 4	Male	31
Donor 5	Female	30	Patient 5	Female	29
Donor 6	Male	41	Patient 6	Male	53
			Patient 7	Male	29
			Patient 8	Male	65
			Patient 9	Female	24
			Patient 10	Female	28
			Patient 11	Male	36
			Patient 12	Female	28

Table S3. Basic information of the HCC patients that had been diagnosed clinically.

HCC Characteristic	Gender	Age	Stage Ⅰ	Stage Ⅱ	Stage Ⅲ	Stage Ⅳ
Patient 1	Female	36	√			
Patient 2	Male	53			√	
Patient 3	Male	35	√			
Patient 4	Female	42		√		
Patient 5	Male	61			√	
Patient 6	Female	63				√
Patient 7	Female	54				√
Patient 8	Male	42		√		
Patient 9	Male	31	√			
Patient 10	Female	35		√		
Patient 11	Female	40		√		
Patient 12	Female	37		√		

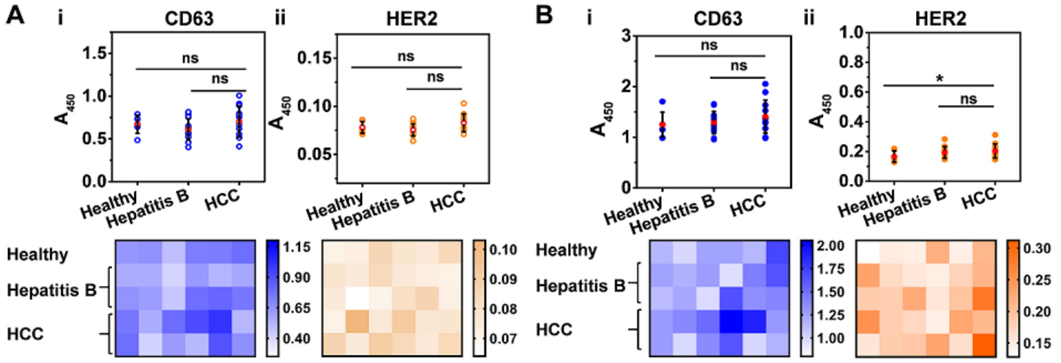

Figure S19. Profiling of CD63 and HER2 in clinical serum samples collected from healthy donors (n = 6), patients with hepatitis B (n = 12), and patients with HCC (n = 12). (i) CD63 and (ii) HER2 profiling were performed using (A) NAISA and (B) ELISA. Heat maps of protein profiles for each sample respectively obtained using (A) NAISA and (B) ELISA. *P < 0.05, ns = no significant difference.

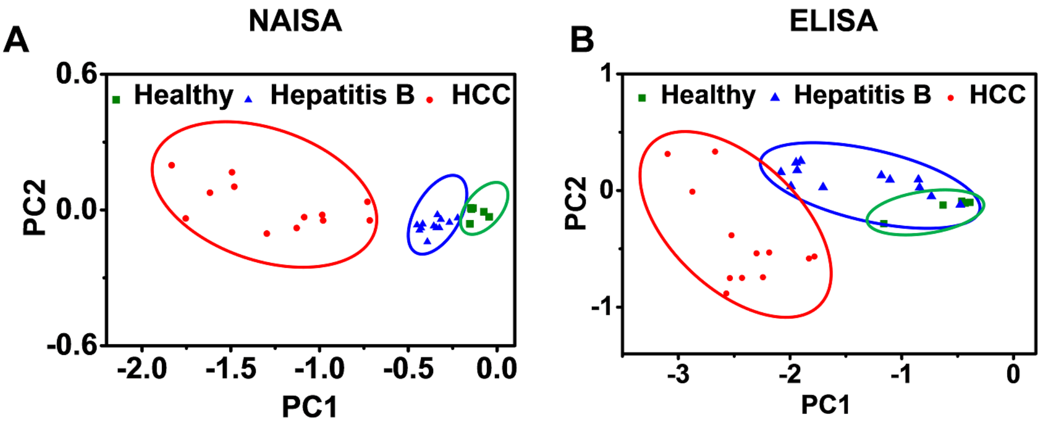

Figure S20. PCA for discrimination of different serum samples by (A) NAISA and (B) ELISA.

Table S4. Comparison of the existing methods for exosomal protein detection.

Methods	Interaction	Specificity	Target signal	Linearity range	Reproducibility	Reference
Aptamer/AuNP biosensor	Protein-aptamer binding	Good	Absorbance	0-12.8 (μg/mL)	Good	15
ELISA	Protein-antibody binding	Good	Absorbance	10^6-10^8 (Paricles /mL)	Good	18
Nano-plasmonic sensor	Protein-antibody binding	Good	Transmission spectra	10^4-10^6 (Paricles /mL)	Medium	18
Microfluidic system	Protein-antibody binding	Good	Fluorescence signal	—	Poor	19
NIR luminescent nanosensor	Protein-aptamer binding	Good	Near-infrared afterglow	—	Medium	22
SERS spectra	Protein-antibody binding	Good	Scattering signals	10^6-10^8 (Paricles /mL)	Medium	25
NAISA	Protein-antibody binding	Good	Absorbance	13.75-220 (μg/mL)	Good	Our method
